# Substance use and substance use disorder, in relation to COVID-19: protocol for a scoping review

**DOI:** 10.1186/s13643-021-01605-9

**Published:** 2021-02-03

**Authors:** Navin Kumar, Kamila Janmohamed, Kate Nyhan, Silvia S. Martins, Magdalena Cerda, Deborah Hasin, Jenny Scott, Richard Pates, Lilian Ghandour, Mayyada Wazaify, Kaveh Khoshnood

**Affiliations:** 1 Human Nature Lab, Department of Sociology, Yale University, New Haven, CT USA; 2 Harvey Cushing/John Hay Whitney Medical Library, Yale University, 333 Cedar Street, New Haven, CT 06520-8014 USA; 3 Department of Environmental Health Sciences, Yale School of Public Health, New Haven, CT, USA, New Haven, CT USA; 4 Department of Epidemiology, Columbia University Mailman School of Public Health, New York, NY USA; 5 Center for Opioid Epidemiology and Policy, Department of Population Health, NYU Grossman School of Medicine, New York, NY USA; 6 Department of Pharmacy and Pharmacology, University of Bath, Bath, UK; 7 Institute of Health and Society, University of Worcester, Worcester, UK; 8 Department of Epidemiology and Population Health, Faculty of Health Sciences, American University of Beirut, Beirut, Lebanon; 9 Department of Biopharmaceuticals and Clinical Pharmacy, School of Pharmacy, University of Jordan, Amman, Jordan; 10 Department of Epidemiology of Microbial Diseases, Yale School of Public Health, New Haven, CT USA

**Keywords:** COVID-19, Substance use disorder, Substance use, SUD

## Abstract

**Background:**

The COVID-19 pandemic is creating severe issues for healthcare and broad social structures, exposing societal vulnerabilities. Among the populations affected by COVID-19 are people engaged in substance use, such as people who smoke; vape (e-cigarette use); use opioids, cannabis, alcohol, or psychoactive prescription drugs; or have a substance use disorder (SUD). Monitoring substance use and SUD during the pandemic is essential, as people who engage in substance use or present with SUD are at greater risk for COVID-19, and the economic and social changes resulting from the pandemic may aggravate SUD. There have been several reviews focused on COVID-19 in relation to substance use and SUD. Reviews generally did not consider on a large range of substance use variants or SUDs. We plan a scoping review that seeks to fill gaps in our current understanding of substance use and SUD, in the COVID-19 era.

**Methods:**

A scoping review focused on substance use and SUD, in relation to COVID-19, will be conducted. We will search (from January 2020 onwards) Cumulative Index to Nursing and Allied Health Literature, Africa-Wide Information, Web of Science Core Collection, Embase, Global Health, WHO Global Literature on Coronavirus Disease Database, WHO Global Index Medicus, PsycINFO, PubMed, Middle Eastern Central Asian Studies, CINAHL Complete, and Sociological Abstracts. Grey literature will be identified using Disaster Lit, Google Scholar, HSRProj, governmental websites, and clinical trials registries (e.g., ClinicalTrial.gov, World Health Organization, International Clinical Trials Registry Platform and International Standard Randomized Con-trolled Trial Number registry). Study selection will conform to Joanna Briggs Institute Reviewers’ Manual 2015 Methodology for JBI Scoping Reviews. Only English language, original studies investigating substance use and SUD, in relation to COVID-19 in all populations and settings, will be considered for inclusion. Two reviewers will independently screen all citations, full-text articles, and abstract data. A narrative summary of findings will be conducted. Data analysis will involve quantitative (e.g., frequencies) and qualitative (e.g., content and thematic analysis) methods.

**Discussion:**

Original research is urgently needed to mitigate the risks of COVID-19 on substance use and SUD. The planned scoping review will help to address this gap.

**Systematic review registration:**

Open Science Framework (osf/io/tzgm5).

**Supplementary Information:**

The online version contains supplementary material available at 10.1186/s13643-021-01605-9.

## Background

The COVID-19 pandemic is creating severe issues for healthcare and broad social structures, exposing societal vulnerabilities [1]. Among the populations affected by COVID-19 are people engaged in substance use, such as people who smoke; vape (e-cigarette use); use opioids, cannabis, alcohol, or psychoactive prescription drugs; or have a substance use disorder (SUD). SUDs are patterns of symptoms resulting from substance use, despite experiencing problems as a result [2]. COVID-19 effects such as lockdowns and social isolation may have also impacted substance use initiation rates.

There may be a relationship between smoking or vaping and susceptibility to SARS-CoV-2 and COVID-19 complications. The percentage of current and former smokers was higher among severe cases of COVID-19 compared to people who never smoked [3, 4]. However, other work indicated that smoking was not related to increased COVID-19 severity [5]. People who vape nicotine or tetrahydrocannabinol may be at risk for COVID-19, with recent work suggesting that vape aerosols may damage lung tissue and reduce lungs’ ability to respond to infections [6].

People currently in treatment for opioid use disorder may be affected by COVID-19 due to reduced access to medication for opioid use disorder (MOUD) [7]. People who receive methadone may face challenges arising from social distancing, as patients usually can only receive a single directly observed daily dose at a time [8], although telemedicine and take home supplies have recently been approved to allow medical providers to start and maintain MOUD [9, 10]. The diversion of resources toward the pandemic may also strain MOUD provision, perhaps leading to patient drop out or medication discontinuation [11, 12]. Social distancing may also increase the possibility of opioid overdoses, with fewer bystanders who can reverse the incident through naloxone administration [1].

Cannabis sales on illicit online markets have risen rapidly during the first 3 months of the pandemic [13]. This may represent an increase in personal use, which may correspond to greater rates of frequent use and cannabis use disorder [14]. Patients with alcohol use disorder (AUD) may also be at greater risk for COVID-19 [15], given that alcohol can weaken the body’s defenses against infections [16]. With social distancing, patients with AUD may have less structured time meant for non-alcohol-related activities [17]. Without structured activities, patients may relapse [15, 18].

Broadly, due to stigma, people with SUD are marginalized and poorly served by healthcare services [1]. Such stigma is persistent even among healthcare workers [19]. If hospitals are resource scarce, people reporting substance use or presenting with SUD may not be the priority if they manifest COVID-19 symptoms, exacerbating the effect of SUD and COVID-19 [1].

There have been several reviews focused on COVID-19, various forms of substance use, and SUD [20–25]. For example, one systematic review detailed COVID-19 and smoking, indicating that smoking may be associated with adverse COVID-19 outcomes [20]. Another review focused on the intersection of alcohol, tobacco, cannabis, opiates, cocaine/crack, and COVID-19 [25]. Reviews generally did not consider a large range of substance use variants or SUDs. Detailing a large range of substance use behaviors and SUDs is key to understanding the broad scope of COVID-19 on possibly marginalized populations. Reviews not centering on less common forms of substance use or SUDs and their relationship with COVID-19 may neglect populations possibly at greater risk during the pandemic. Overall, monitoring substance use and SUD during the pandemic is essential, as people who engage in substance use, or present with SUD, may be at greater risk for COVID-19, and the economic and social changes resulting from the pandemic may be the course of SUD [26].

The planned scoping review will synthesize and determine research priorities around COVID-19, substance use and SUD, by providing information that can be used to develop interventions around substance use and SUD, in relation to COVID-19, and also identify gaps in research. We will conduct a scoping review rather than use other methods of research synthesis because scoping reviews are appropriate for mapping an area of research [27]; we will not be examining the effect of an intervention on an outcome of interest, and it thus does not make sense to assess risk of bias, as per a systematic review, and SUD and substance use research outcomes are likely not sufficiently similar to each other to warrant pooling or formal meta-analysis regarding a specific outcome. Key to the development of interventions that mitigate negative substance use and SUD-related outcomes amid COVID-19 is a comprehensive understanding of the current status of evidence around substance use and SUD during the COVID-19 era. The planned scoping review seeks to understand gaps in the current knowledge base by contributing an evaluation of what is currently known about substance use and SUD in relation to COVID-19. Past work has focused on a limited range of substance use variants and SUDs, and the planned review will expand to a broader range of substance use types and SUDs.

## Methods/design

The review protocol has been registered within the Open Science Framework database (osf/io/tzgm5) and is being reported in accordance with the reporting guidance provided in the Preferred Reporting Items for Systematic Reviews and Meta-Analyses Protocols (PRISMA-P) statement [28] (see checklist in Additional file 1). The proposed scoping review will be reported in accordance with the reporting guidance provided in the Preferred Reporting Items for Systematic Reviews and Meta-analyses (PRISMA) extension for Scoping Reviews (PRISMA-ScR) [29]. Research objectives, inclusion criteria, and methodological techniques will be determined before study commencement using the Joanna Briggs Institute Reviewers’ Manual 2015 Methodology for JBI Scoping Reviews [30]. This process will adhere to the indicated framework: (1) identifying research question; (2) developing comprehensive search strategy; (3) identifying relevant studies; (4) selecting studies; (5) charting data; and (6) collating, summarizing, and reporting results. The study team will develop a search strategy as recommended by the 2015 Methodology for JBI Scoping Reviews.

This scoping review will be conducted by 11 individuals: 10 researchers from several universities worldwide, from a range of disciplines (e.g., public health, economics, epidemiology, and pharmacy), and an informationist from the Harvey Cushing/John Hay Whitney Medical Library at Yale University. The objective of the scoping review is to develop a better understanding of the current research landscape around SUDs and substance use and COVID-19 by investigating existing studies and gaps in the research. The broad research questions are “What does current research suggest about the impact of substance use and SUDs on COVID-19 infection and progression?” and “What impact has COVID-19 had on substance use and SUD rates?” The search strategy will be performed in line with techniques that enhance methodological transparency and improve the reproducibility of the results and evidence synthesis.

### Information sources and search strategy

The primary source of literature will be a structured search of electronic databases (from January 2020 onwards): Cumulative Index to Nursing and Allied Health Literature, Africa-Wide Information, Web of Science Core Collection, Embase, Global Health, WHO Global Literature on Coronavirus Disease Database, WHO Global Index Medicus, PsycINFO, PubMed, Middle Eastern Central Asian Studies, CINAHL Complete, and Sociological Abstracts. The secondary source of potentially relevant material will be a search of preprint servers (e.g., medRxiv.org, PsyArXiv.org), Disaster Lit, Google Scholar (e.g., the first five pages will be searched), governmental websites, and clinical trials registries (e.g., ClinicalTrial.gov, World Health Organization International Clinical Trials Registry Platform and International Standard Randomized Controlled Trial Number registry, HSRProj). The references of included documents will be hand-searched to identify any additional evidence sources. The search strategy will be designed by a research librarian and peer reviewed by using the Peer Review of Electronic Search Strategies (PRESS) checklist [31]. A draft search strategy for MEDLINE is provided in Additional file 2. We will use search terms similar to our main search to find articles for inclusion. The same keywords for the main search will be used to search grey literature each time. All grey literature will be compiled in a folder and reviewed similarly to articles obtained from our database searches. EndNote, a bibliographic software, will be used to store, organize, and manage all references [32].

### Eligibility criteria

We will include all studies with all study designs involving substance use and SUD, in relation to COVID-19. Only English language studies will be considered for inclusion. Past work indicated that excluding non-English language records from a review seemed to have a minimal effect on results [33, 34].

#### Inclusion criteria

Published research (peer reviewed and grey literature where primary data was collected such as reports, research letters and briefs) investigating substance use and SUD, in relation to COVID-19 in all populations, settings, and study designs, e.g., studies with small samples, quantitative, and qualitative studies, will be eligible for inclusion. All variants of substance use disorder will be included, such as opioid use disorder, cannabis use disorder, and alcohol use disorder. All variants of substance use, smokable or otherwise, will be included, such as alcohol, tobacco, nicotine, cannabis, cocaine, methamphetamine, non-medical use of psychoactive prescription drugs, and opioids.

There will be no restrictions on age, region, or gender.

Studies reported only as conference abstracts will be included, only if we do not have access to the full paper. Conference abstracts are often left out of systematic reviews as they may not contain adequate information to conduct quality assessment or a meta-analysis. Here, we will include conference abstracts as they are often published earlier than full manuscripts [35], which is key to a thorough scoping review on an ongoing phenomenon.

#### Exclusion criteria

Commentaries, correspondences, case reports, case series, editorials, and opinion pieces will be excluded. Case reports and case series often contain relatively limited evidence [36].

Governmental or other agency guidelines will be excluded.

Reviews such as systematic reviews and scoping reviews will be excluded, but we will review the references in these for inclusion, if applicable.

### Screening and selection procedure

All reports identified from the searches will be screened by two reviewers independently. First, titles and abstracts of articles returned from initial searches will be screened based on the eligibility criteria outlined above. Second, full texts will be examined in detail and screened for eligibility. Third, references of all considered articles will be hand-searched to identify any relevant report missed in the search strategy. Any disagreements will be resolved by discussion, or if necessary, with a third reviewer. A flow chart showing details of studies included and excluded at each stage of the study selection process will be provided. We will contact authors where necessary if the abstracts do not provide sufficient information [35]. Covidence will be used to manage the title/abstract and full-text screening phases [37].

### Data extraction

Reviewers will undergo a practice exercises till they have a high level of agreement (*>*0.8 kappa) and then independently extract data from studies. Reviewers will abstract the data using a pretested data extraction template. We will use a standardized coding protocol to collect information such as title of study; authors; date published; author affiliation as a measure to ascertain the discipline focus of the study and collaborating institutions; study setting; study design; description of methodology; description of study sample; definition or type of substance/SUD studied (if any); measurements and scales used; main findings; funder information; journal title; and submission variant (research letter, short report, original article etc.). Even though a formal risk of bias is not planned for this scoping review, we will note which studies are pre-prints and, thus, have not been formally peer reviewed.

### Data synthesis

Outcomes and other information collected regarding selected studies will be synthesized using quantitative (e.g., frequencies) and qualitative (e.g., content and thematic analysis) methods, with a narrative summary of findings conducted. Synthesis will be presented in tables, summary data in graphs, and individual data for each study in tables. The broad goal of the synthesis is to identify gaps in research and present recommendations for future research agendas.

## Discussion

The strength of the planned scoping review is the use of a transparent and reproducible procedure for a scoping literature review. We state the data sources, search strategy, and data extraction [38]. Through publishing this research protocol, we strengthen the clarity of the search strategy.

There have been few studies which compile available evidence from various settings around substance use and SUD, in relation to COVID-19. Our review will provide an overview of these studies, synthesizing evidence. There is much anecdotal work around substance use and SUD, in relation to COVID-19, with few published studies. The planned review will highlight areas of research focus and gaps which require more attention. Moreover, the COVID-19 context is quickly changing [39] likely affecting SUDs and substance use in a rapidly shifting fashion. Results will thus provide high-level information to inform, support, and customize design of interventions to mitigate reduced health outcomes in this setting. As researchers attempt to minimize the harms from COVID-19, they need to be aware of scientific evidence to develop interventions to achieve their aim. The planned scoping review seeks to provide this evidence by contributing an evaluation of what is currently known about substance use and SUD, in relation to COVID-19, with the goal of identifying gaps in research and presenting recommendations for future research foci.

Any amendments to this protocol will be documented in the final published scoping review with reference to saved searches and analysis.

Results of the review will be disseminated in a peer-reviewed journal and likely in other media such as conferences, seminars, and symposia. The protocol and final review article will be made open access upon publication. As per PRISMA-ScR guidelines, we will present results in a user-friendly format [40].

### Limitations

Our planned review should be read in line with some limitations. Although we plan to search several databases and grey literature sources, we may miss some studies. Not all authors we reach out to may respond and we may thus miss some unpublished work. We may not be able to make policy recommendations due to the lack of quality appraisal of studies [41].

## Supplementary Information

**Additional file 1.** Preferred Reporting Items for Systematic reviews and Meta-Analyses extension for Scoping Reviews (PRISMA-ScR) Checklist

**Additional file 2.** Draft search strategy for MEDLINE

## Data Availability

The datasets used and analyzed will be made available upon reasonable request.

## Abbreviations

AUD: Alcohol use disorder
MOUD: Medication for opioid use disorder
SUD: Substance use disorder
JBI: Joanna Briggs Institute
COVID-19: Coronavirus Disease 2019
SARS-CoV-2: Severe acute respiratory syndrome coronavirus 2
