# Supplementary material for: Substance use and substance use disorder, in relation to COVID-19: protocol for a scoping review
Source: Syst Rev. 2021 Feb 3;10:48. doi: 10.1186/s13643-021-01605-9 (PMC7857102; doi:10.1186/s13643-021-01605-9)
Supplement: Supplementary file 2 — Additional file 2. Draft search strategy for MEDLINE [file 13643_2021_1605_MOESM2_ESM.docx]

# MEDLINE DRAFT SEARCH STRATEGY

**Search strategy**

1. exp Coronavirus/
2. exp Coronavirus Infections/
3. (coronavirus* or corona virus* or OC43 or NL63 or 229E or HKU1 or HCoV* or ncov* or covid* or sars-cov* or sarscov* or Sars-coronavirus* or Severe Acute Respiratory Syndrome Coronavirus*).mp.

4 (or/1-3) and ((20191* or 202*).dp. or 20190101:20301231.(ep).) [this set is the sensitive/broad part of the search]

1. 4 not (SARS or SARS-CoV or MERS or MERS-CoV or Middle East respi- ratory syndrome or camel* or dromedar* or equine or coronary or coronal or covidence* or covidien or influenza virus or HIV or bovine or calves or TGEV or feline or porcine or BCoV or PED or PEDV or PDCoV or FIPV or FCoV or SADS-CoV or canine or CCov or zoonotic or avian influenza or H1N1 or H5N1 or H5N6 or IBV or murine corona*).mp. [line 5 removes noise in the search results]
2. ((pneumonia or covid* or coronavirus* or corona virus* or ncov* or 2019-ncov or sars*).mp. or exp pneumonia/) and Wuhan.mp.
3. (2019-ncov or ncov19 or ncov-19 or 2019-novel CoV or sars-cov2 or sars-cov-2 or sarscov2 or sarscov-2 or Sars-coronavirus2 or Sars-coronavirus-2 or SARS-like coronavirus* or coronavirus-19 or covid19 or covid-19 or covid 2019 or ((novel or new or nouveau) adj2 (CoV or nCoV or covid or coronavirus* or corona virus or Pandemi*2)) or ((covid or covid19 or covid-19) and pandemic*2) or (coronavirus* and pneumonia)).mp.
4. COVID-19.rx,px,ox. or severe acute respiratory syndrome coronavirus 2.os.

9 (”32240632” or ”32236488” or ”32268021” or ”32267941” or ”32169616” or

”32267649” or ”32267499” or ”32267344” or ”32248853” or ”32246156” or

”32243118” or ”32240583” or ”32237674” or ”32234725” or ”32173381” or

”32227595” or ”32185863” or ”32221979” or ”32213260” or ”32205350” or

”32202721” or ”32197097” or ”32196032” or ”32188729” or ”32176889” or

”32088947” or ”32277065” or ”32273472” or ”32273444” or ”32145185” or

”31917786” or ”32267384” or ”32265186” or ”32253187” or ”32265567” or

”32231286” or ”32105468” or ”32179788” or ”32152361” or ”32152148” or

”32140676” or ”32053580” or ”32029604” or ”32127714” or ”32047315” or

”32020111” or ”32267950” or ”32249952” or ”32172715”).ui.

10 or/6-9 [Lines 6 to 9 are specific to Covid-19] 11 5 or 10

12 11 and 20191201:20301231.(dt).

1. (covid* or ncov* or 2019-novel CoV or SARS-CoV2 or SARS-CoV-2 or SARSCoV2 or SARSCov-2 or ”severe acute respiratory syndrome coronavirus 2”).mp.
2. (coronavirus* or corona virus*).mp. and 2020*.dp.
3. ((novel or new or ”2019” or ”19” or pandemic or crisis or outbreak or Wuhan or China) adj3 (coronavirus* or corona virus*)).mp.
4. Covid-19.rx.

23642

1. coronavirus infections/ and 2020*.dp.
2. Pneumonia, Viral/ and 2020*.dp.

19 or/13-18

58329

20 12 or 19

58413

1. [from the CADTH hedge]
2. (coronavirus/ or betacoronavirus/ or coronavirus infections/) and (disease out- breaks/ or epidemics/ or pandemics/)
3. (nCoV* or 2019nCoV or 19nCoV or COVID19* or COVID or SARS- COV-2 or SARSCOV-2 or SARSCOV2 or Severe Acute Respiratory Syn- drome Coronavirus 2 or Severe Acute Respiratory Syndrome Corona Virus 2).ti,ab,kf,nm,ot,ox,rx,px.
4. ((new or novel or ”19” or ”2019” or Wuhan or Hubei or China or Chi- nese) adj3 (coronavirus* or corona virus* or betacoronavirus* or CoV or HCoV)).ti,ab,kf,ot.
5. ((coronavirus* or corona virus* or betacoronavirus*) adj3 (pandemic* or epi- demic* or outbreak* or crisis)).ti,ab,kf,ot.
6. ((Wuhan or Hubei) adj5 pneumonia).ti,ab,kf,ot. 27 or/22-26
7. limit 27 to yr=”2019 -Current”
8. 20 [homegrown]
9. 27 [CADTH] 31 20 or 27
10. (epidemic or pandemic or crisis or social* distan* or quarantin* or self-isolat* or recession*).mp. or economic recession/
11. [substance use]
12. [opioid use]
13. substance-related disorders/ or opioid-related disorders/ or heroin dependence/ or morphine dependence/ or opium dependence/ or substance abuse, intra- venous/ or substance abuse, oral/ or substance withdrawal syndrome/ or methadone/ or opiate substitution treatment/
14. ((opioid* or opiate* or heroin or amphetamine*) adj3 (abus* or dependen* or disorder* or addict* or misus* or ”use” or ”user” or ”users” or ”usage” or ”using” or ”used”)).mp.
15. exp Narcotics/po
16. (OUD or IDU or PWID).mp.
17. (”injection drug use” or ”intravenous drug use”).mp.
18. [smoking and vaping]
19. exp smoking/
20. ”tobacco use disorder”/
21. smoking cessation/
22. ”tobacco use cessation”/
23. vaping/
24. Electronic Nicotine Delivery Systems/
25. marijuana abuse/ or exp ”marijuana use”/
26. exp tobacco prod- ucts/
27. (smoking or smoker* or cigarette* or ecig* or e-cig* or tobacco or snuff or snus or cannabis or marijuana or vape or vaping or vaper or vapers or vaped or cigar*).mp.
28. [alcohol]
29. exp alcohol-related disorders/
30. (alcohol* or ”alcohol use” or alcohol abuse* or wine* or beer* or liquor* or spirits).mp.
31. [big picture]
32. exp substance-related disorders/ or illicit drugs/
33. ((substance* adj3 (abus* or dependen* or disorder* or addict* or misus* or ”use” or ”user” or ”users” or ”usage” or ”using” or ”used”)) or addiction* or addict*).mp.
34. [additional substances]
35. aerosol propellant*.mp.
36. ((sniff* or huff* or inhal*) adj2 aerosol*).mp.
37. inhalant abuse.mp.
38. huffing.mp.
39. aliphatic nitrites.mp.
40. (amphetamine* or speed).mp.
41. anabolic steroid*.mp.
42. doping in sports/ and exp steroids/
43. performance-enhancing substances/
44. (phencyclidine or PCP or angel dust).mp.
45. exp Benzodiazepines/ae
46. benzodiazepine*.mp.
47. cocaine.mp.
48. (ecstasy or MDMA).mp.
49. exp Hallucinogens/ae
50. N-Methyl-3,4-methylenedioxyamphetamine/
51. (GBH or GBL or gamma-hydroxybutyrate).mp.
52. 4-Butyrolactone/
53. glue.mp.
54. (hashish or heroin).mp.
55. exp ”hypnotics and sedatives”/
56. hypnotics.mp.
57. inhalant*.mp.
58. (LSD or Lysergic Acid Diethylamide).mp.
59. mescaline/
60. mescaline.mp.
61. exp amphetamines/
62. methamphetamine*.mp.
63. methylxanthine*.mp.
64. nicotine.mp. or nicotine chewing gum/ or nicotine/
65. (nitrous oxide or laughing gas).mp.
66. ((OTC or over the counter) adj1 (drug* or medication*)).mp.
67. nonprescription drugs/ae
68. oxycodone.mp.
69. paint thinner*.mp.
70. amyl nitrite.mp.
71. poppers.mp.
72. psilocybe.mp.
73. salvia divinorum.mp.
74. sedative*.mp.
75. stimulant*.mp.
76. tranquilizer*.mp.
77. designer drugs/ 100 or/33-99

101 100 and (31 or 32)

1. limit 101 to (english language and yr=”2020 -Current”)
2. [compare with the similar search, without line 32] 104 100 and 31

1373

1. limit 104 to (english language and yr=”2020 -Current”)
2. [the difference] 107 102 not 105
